# Laparoscopic Resection of a Diaphragmatic Phrenic Neurilemmoma Compressing the Suprahepatic Inferior Vena Cava Following Thoracoscopic Exploration: A Case Report

**DOI:** 10.70352/scrj.cr.25-0678

**Published:** 2026-01-06

**Authors:** Masashi Tsunematsu, Koichiro Haruki, Ryoga Hamura, Norimitsu Okui, Shinji Onda, Taro Sakamoto, Tomohiko Taniai, Kenei Furukawa, Jungo Yasuda, Toru Ikegami

**Affiliations:** Division of Hepatobiliary Pancreatic Surgery, Department of Surgery, The Jikei University School of Medicine, Tokyo, Japan

**Keywords:** diaphragm, neurilemmoma, suprahepatic vena cava

## Abstract

**INTRODUCTION:**

Surgical resection of tumors compressing the suprahepatic inferior vena cava (IVC) is challenging, as such lesions may require vascular resection, reconstruction, or extracorporeal circulation. We present a case of a diaphragmatic phrenic neurilemmoma severely compressing the suprahepatic IVC and right hepatic vein, which was successfully resected laparoscopically with both thoracoscopic and laparoscopic assessment.

**CASE PRESENTATION:**

A 54-year-old woman was referred for evaluation of a thoracic mass detected during a health check-up. Enhanced CT revealed a 2.7-cm, well-circumscribed, heterogeneously enhancing round tumor compressing the suprahepatic IVC and right hepatic vein. Thoracoscopic exploration suggested that the tumor was not intrathoracic, and the right diaphragmatic nerve was identified near the lesion. Laparoscopic resection was then performed with preparation for possible open conversion. After establishing pneumoperitoneum, 4 additional ports were inserted. Dissection of the falciform and coronary ligaments exposed a well-encapsulated tumor originating from the diaphragm. The inferior diaphragmatic vein was transected using ultrasonic shears. The tumor was carefully dissected from the diaphragm without invasion into the IVC or hepatic vein. Complete resection was achieved without removal of adjacent organs, including the diaphragm. The specimen was retrieved via the umbilical incision. Operative time was 54 min, and blood loss was 2 mL. The postoperative course was uneventful. Histopathology revealed benign spindle cells arranged in a storiform pattern, confirming a benign neurilemmoma.

**CONCLUSIONS:**

Laparoscopic resection of diaphragmatic phrenic neurilemmoma compressing the suprahepatic IVC can be safe and feasible when combined with careful intraoperative assessment. Thoracoscopic evaluation and preparation for potential vascular involvement are crucial to guide safe resection and manage possible adhesion or invasion.

## Abbreviation

IVC
: inferior vena cava

## INTRODUCTION

Surgical resection of tumors compressing the suprahepatic inferior vena cava (IVC) can be challenging, as such lesions may require vascular resection, reconstruction, or the use of extracorporeal circulation.^1,2)^ Tumors originating from the diaphragm are particularly rare and may be difficult to distinguish from hepatic or retroperitoneal masses preoperatively. In addition to their rarity, lesions arising from the diaphragm often present a diagnostic challenge because conventional imaging alone may not clearly differentiate whether the tumor is located in the thoracic or abdominal cavity. Accurate identification of the tumor’s origin is crucial for determining the optimal surgical approach, particularly when the lesion lies adjacent to major vessels such as the suprahepatic IVC or hepatic veins.

Herein, we report a case of a diaphragmatic phrenic neurilemmoma that was successfully resected laparoscopically, with thoracoscopic observation playing a pivotal role in determining the tumor’s true origin and informing the subsequent laparoscopic procedure.

## CASE PRESENTATION

**Fig. 1:**
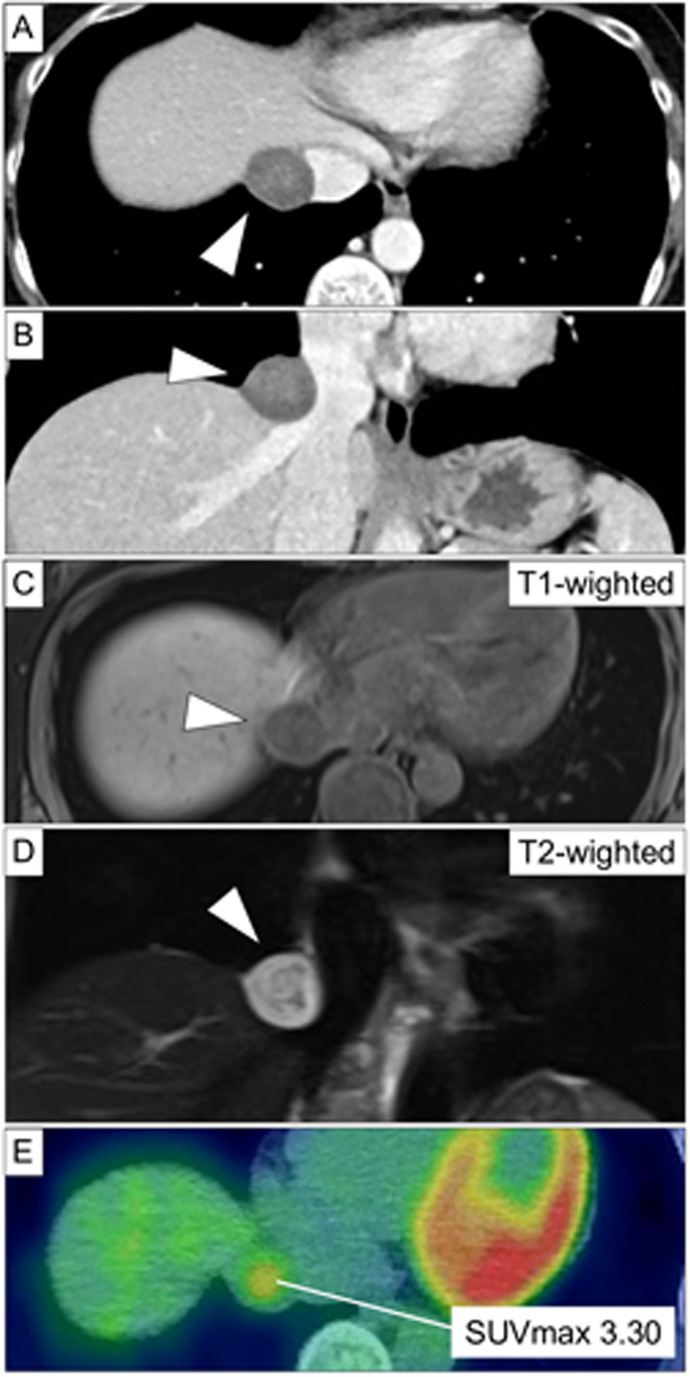
CT demonstrates a well-circumscribed but heterogeneously enhancing round tumor (arrowhead) compressing the suprahepatic inferior vena cava and right hepatic vein (**A**, **B**). MRI demonstrated a lesion (arrowhead) with high peripheral signal intensity on T2-weighted imaging, heterogeneous low internal intensity, and homogeneous signal intensity comparable to skeletal muscle on T1-weighted imaging (**C**, **D**). FDG-PET revealed mild uptake with a maximum standardized uptake value of 3.30 (**E**).

A 54-year-old woman was referred for evaluation of a thoracic mass detected during a routine health check-up. The lesion, initially diagnosed as a benign cystic tumor in the thoracic cavity, had increased in size over 2 years. Enhanced CT revealed a 2.7-cm, well-circumscribed, heterogeneously enhancing round tumor that severely compressed the suprahepatic IVC and right hepatic vein (**Fig. 1A** and **1B**). MRI demonstrated a lesion with high peripheral signal intensity on T2-weighted imaging, heterogeneous low internal intensity, and homogeneous signal intensity comparable to skeletal muscle on T1-weighted imaging, without diffusion restriction (**Fig. 1C** and **1D**). FDG-PET revealed mild uptake with a maximum standardized uptake value of 3.30 (**Fig. 1E**). No abnormalities were detected on blood tests, including tumor markers. Based on these findings, a neurilemmoma was considered the most likely diagnosis, although other neurogenic tumors and solitary fibrous tumor were also included in the differential diagnoses. Because imaging could not clearly determine whether the tumor originated from the thoracic cavity, diaphragm, or liver, thoracoscopic exploration was planned first to clarify its anatomical location and assess the feasibility of thoracic resection (**Supplementary Video**).

**Fig. 2:**
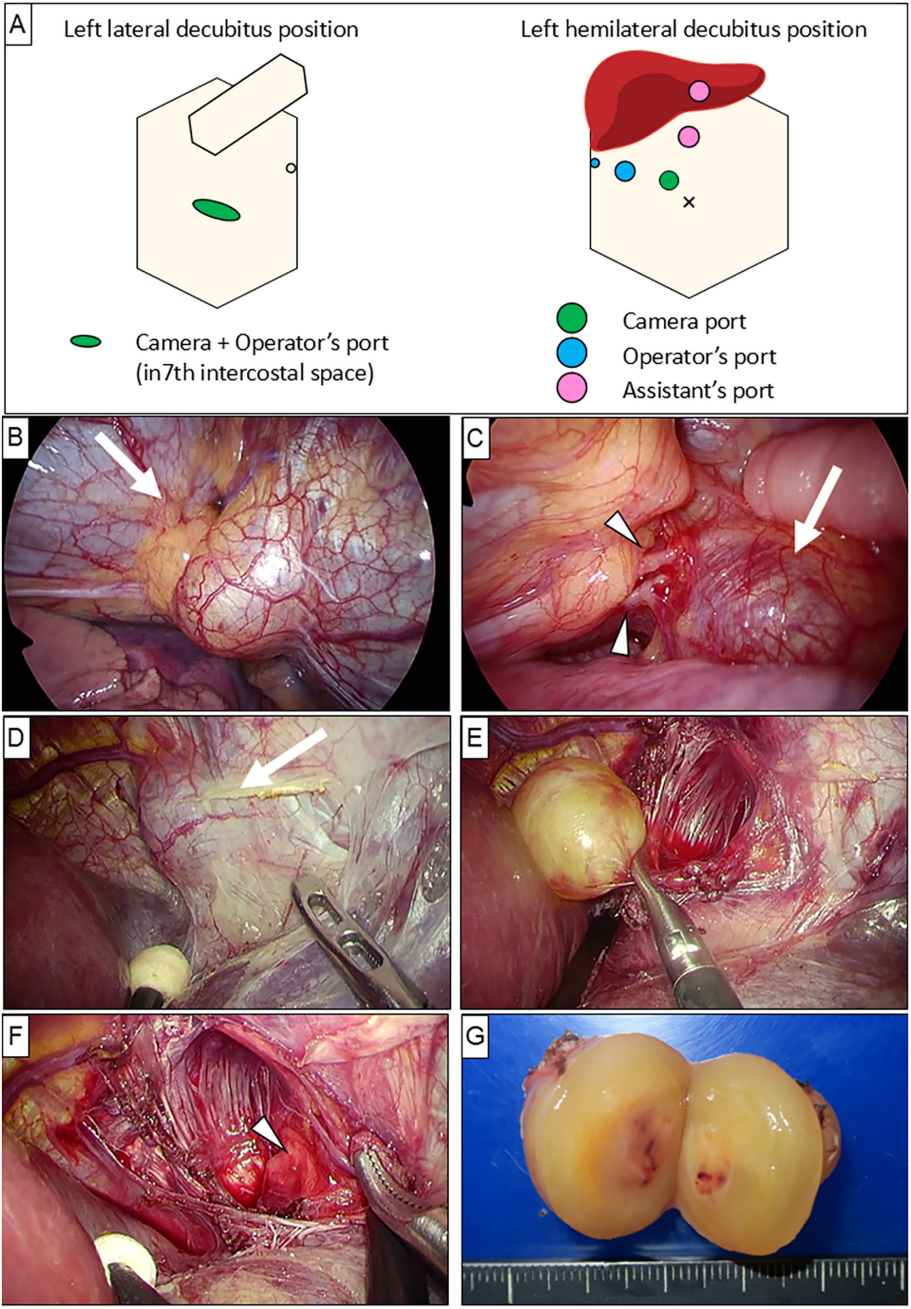
Port placement for thoracoscopy and laparoscopy (**A**). Thoracoscopy revealed the tumor (arrow) was covered by the central tendon of the diaphragm (**B**). The right diaphragmatic nerve (arrowheads) was found near the tumor (arrow) (**C**). Laparoscopic dissection of the coronary ligament showed a completely encapsulated tumor originating from the diaphragm (arrow) (**D**). The tumor was carefully dissected from the diaphragm (**E**). The tumor did not invade the suprahepatic inferior vena cava (arrowhead) (**F**). The resected specimen (**G**).

Thoracoscopy was performed with the patient in the left lateral decubitus position, using a 30° thoracoscope introduced through the 7th intercostal space at the mid-axillary line (**Fig. 2A**). Thoracoscopic exploration revealed that the tumor was completely covered by the central tendon of the diaphragm, suggesting an abdominal origin (**Fig. 2B**). The right phrenic nerve was identified near the lesion (**Fig. 2C**). These findings suggested that the lesion originated from the abdominal rather than thoracic side; therefore, laparoscopic resection was subsequently performed.

With the patient in the supine position, the first camera port was inserted using an open technique, followed by 4 additional ports after establishing pneumoperitoneum (**Fig. 2A**). Dissection of the falciform and coronary ligaments exposed a well-encapsulated tumor arising from the diaphragm (**Fig. 2D**). The inferior diaphragmatic vein was divided using ultrasonic shears. The tumor was carefully dissected from the diaphragm and separated from the suprahepatic IVC and right hepatic vein without evidence of direct tumor invasion (**Fig. 2E**). Complete resection was achieved without removal of other organs, including the diaphragm (**Fig. 2F**).

The specimen was retrieved using a reinforced laparoscopic retrieval bag via the umbilical incision. The operative time was 54 min, and blood loss was 2 mL. The postoperative course was uneventful, and the patient was discharged on POD 5.

Macroscopically, the tumor was a multilobulated, yellowish solid mass (**Fig. 2G**). Histopathological examination revealed benign spindle cells with a storiform pattern, consistent with a benign neurilemmoma. Based on both thoracoscopic and laparoscopic findings, the tumor was considered to originate from the peripheral phrenic nerve.

## DISCUSSION

The present case highlights the clinical significance of combined thoracoscopic and laparoscopic evaluation for tumors located at the thoracoabdominal junction, especially when imaging studies cannot clearly distinguish whether the lesion arises from the thoracic cavity, diaphragm, or abdominal structures. This approach allowed precise intraoperative identification of the tumor origin from the peripheral branch of the right phrenic nerve, as well as its spatial relationship to the suprahepatic IVC and right hepatic vein, guiding a safe minimally invasive resection.

Preoperative imaging with contrast-enhanced CT or MRI may not always clearly distinguish the diaphragmatic origin from adjacent structures. In this case, thoracoscopy provided several strategic advantages. First, it allowed direct visualization of the central tendon and confirmed that the lesion did not arise from the thoracic cavity. Second, identification of the right phrenic nerve adjacent to the lesion strongly suggested a peripheral phrenic nerve origin. These intraoperative findings prevented an unnecessary thoracic approach and guided a safe transition to laparoscopic resection. Without thoracoscopic evaluation, the operative plan might have involved a more invasive thoracic procedure or uncertainty regarding the feasibility of laparoscopic resection.

Once the tumor origin was clarified, laparoscopy offered excellent magnified visualization for careful dissection along the diaphragm and major vessels. Although diaphragmatic neurilemmomas are usually benign and noninvasive,^3,4)^ their proximity to the suprahepatic IVC and hepatic veins necessitates meticulous surgical preparation.^5,6)^ Importantly, we equipped the operating field to allow immediate open conversion if unexpected dense adhesions or vascular involvement were encountered. In our case, careful preoperative planning and precise dissection enabled complete resection of the tumor without diaphragmatic resection, vascular injury, or conversion to open surgery. The combination of thoracoscopic and laparoscopic observations was instrumental in securing an optimal operative field and preventing complications.^7)^

Although laparoscopic resection of diaphragmatic neurilemmomas has been reported, few cases include both thoracoscopic and laparoscopic intraoperative findings. Presenting both views highlights the value of multidisciplinary planning and careful assessment. While combined thoracoscopic and laparoscopic approaches are not always required, they can be particularly useful when the tumor origin is unclear on preoperative imaging. This experience suggests that, with appropriate planning and technical consideration, minimally invasive approaches can be safely applied to diaphragmatic neurilemmomas compressing major vessels.

## CONCLUSIONS

Laparoscopic resection of diaphragmatic phrenic neurilemmoma compressing the suprahepatic IVC can be safe and feasible when combined with careful intraoperative assessment. Thoracoscopic evaluation and preparation for potential vascular involvement are crucial to guide safe resection and manage possible adhesion or invasion.

## SUPPLEMENTARY MATERIAL

Supplementary video Preoperative enhanced CT showed a round tumor that severely compressed the suprahepatic IVC and right hepatic vein. Laparoscopic observation revealed a completely encapsulated tumor originating from the diaphragm. Complete surgical resection of the encapsulated tumor without partial resection of other organs, including the surrounding diaphragm, was performed.

## DECLARATIONS

### Availability of data and materials

The data that support the findings of this study are available from the corresponding author upon reasonable request.
